# Strict Surgical Repair for Bile Leakage Following the Roux-en-Y Hepaticojejunostomy

**DOI:** 10.3389/fsurg.2021.641127

**Published:** 2021-05-04

**Authors:** Keying Zhang, Linfeng Wu, Kai Gao, Chengwei Yan, Chao Zheng, Chunbao Guo

**Affiliations:** ^1^College of Traditional Chinese Medicine, Chongqing Medical University, Chongqing, China; ^2^Department of Pediatric General and Neonatal Surgery, Children's Hospital of Chongqing Medical University, Chongqing, China; ^3^Ministry of Education Key Laboratory of Child Development and Disorders, Children’s Hospital of Chongqing Medical University, Chongqing, China; ^4^Department of Orthopaedics, Children’s Hospital of Chongqing Medical University, Chongqing, China; ^5^China International Science and Technology Cooperation Base of Child Development and Critical Disorders, Children’s Hospital of Chongqing Medical University, Chongqing, China; ^6^Chongqing Key Laboratory of Pediatrics, Children’s Hospital of Chongqing Medical University, Chongqing, China; ^7^Chongqing Engineering Research Center of Stem Cell Therapy, Children’s Hospital of Chongqing Medical University, Chongqing, China; ^8^Department of Pediatric General Surgery, Sanxia Hospital, Chongqing University, Chongqing, China; ^9^Department of Traumatology, Children’s Hospital of Chongqing Medical University, Chongqing, China

**Keywords:** Roux-en-Y hepaticojejunostomy, bile leakage, redo surgery, strict surgical repair, children

## Abstract

**Background:** The optimal bile leakage management strategy in the pediatric population following the initial Roux-en-Y hepaticojejunostomy is still a matter of discussion today. Here, we assessed the roles of bile leakage management and surgical implementation on outcomes for patients with bile leakage.

**Materials and Methods:** A revised protocol for bile leakage management with restricted surgical intervention was implemented at Chongqing Children’s Hospital on March 15, 2013 and Sanxia Hospital on April 20, 2013. We performed a retrospective, historical control analysis for the protocol implementation to compare the short- and long-term outcomes using the corresponding statistical methods.

**Results:** There was a total of 84 patients included in the analysis, including 46 patients in the pre-protocol group and 38 patients in the post-protocol group. No statistical differences for the demographic features were found between the two groups. There was a decrease in redo surgeries in the post-protocol cohort compared to those in the pre-protocol cohort (odds ratio [OR] = 4.48 [95% CI, 1.57–12.77]; *p* = 0.003). Furthermore, patients in the post-protocol group were less likely to be associated with intensive care unit (ICU) admission (OR = 3.72 [95% CI, 1.11–12.49]; *p* = 0.024) compared to patients in the pre-protocol group, respectively. There was no mortality between the two groups.

**Conclusions:** A restrictive surgical intervention strategy can effectively reduce the rate of redo surgery and exhibited promising outcomes for bile leakage in terms of postoperative recovery and hospitalization costs.

## Introduction

The preferred management for choledochal cysts is cyst excision with biliary reconstruction for hepaticojejunostomy. Despite improvements in operative skill and perioperative care, short- and long-term complications still remain common in children, among them, bile leakage is a major source of concern (1–3). Over the past decade, the incidence of bile leakage following biliary-enteric anastomosis for choledochal cyst was still high, reportedly ranging from 2.3 to >10% (4, 5).

The development of bile leakage may affect late functional recovery, as well as the occurrence of biliary stricture (6, 7). The management of bile leakage usually varied by the individual surgeon or institutional philosophy from conservation management to a multidisciplinary approach, such as, endoscopic dilatation, percutaneous drainage, etc., Leakage with severe clinical manifestations or failure of the multidisciplinary approach generally needs redo surgery (8). However, it is a matter of debate on the timing of the surgical intervention and not enough high-quality evidence to either encourage or discourage the surgical treatment of this troublesome problem, and so the optimal strategy for bile leakage is still debated (9–12).

In our institute, the patients have been managed with individual experience. We noticed a need for multidisciplinary management based primarily on the condition of the patient. However, this has only been spuriously investigated. On 2013, we proposed a restrictive protocol for bile leakage management, based largely on the present references. The present study aimed to explore whether the new protocol would lead to a decrease redo operations for pediatric patients. We also evaluated the postoperative and long-term outcomes after the implementation of this protocol.

## Materials and Methods

### Patient Characteristics

The retrospective analysis of the patients with bile leakage following elective Roux-en-Y hepaticojejunostomy from January 2004 to March 2020 in a collaborative multidisciplinary program among three pediatric general surgical departments at Qingdao Maternity and Child Care Hospital, Sanxia Hospital, Chongqing University, and Chongqing Children’s Hospital. Review Board (609/2020) of the Chongqing Children’s Hospital, Chongqing Medical University. All the procedures among these patients were performed by five attending surgeons, and no differences were noted among all of the surgical features of these surgeons. An abdominal drainage tube was routinely placed next to the anastomosis site.

### The Bile Leakage Evaluation

In our institution, postoperative bile leakage is comprehensively diagnosed at the time of initial presentation. Clinical physical examination, laboratory testing, imaging studies (abdominal ultrasonography or MRCP), and occasionally computed tomography were carried out to verify the diagnosis of bile anastomotic leakage. The symptoms ranged from an asymptomatic leak to a leak resulting in a life-threatening condition. The general postoperative signs included abdominal pain, abdominal distension, dehydration, fever, and electrolyte disturbances, as well as peritonitis. More specific signs suggestive of a bile leak are the increased total bilirubin concentrations in drain fluid. Since 2012, bile leakage was defined biochemically by a bilirubin ratio of the drain fluid to serum of at least 3.0 on or after postoperative day 3 based on the criteria of the International Study Group of Liver Surgery (ISGLS) (9).

The volume of bile leakage was another important concern, which might determine the clinical treatment.

### Protocol Implementation

Because standard evidence for the management of bile leakage was unavailable, we usually implemented the empiric perioperative management practices for the patients with bile leakage following Roux-en-Y hepaticojejunostomy. After 2013, we upgraded this criteria to a new protocol. In the post-protocol management, the difference was the implementation of the restrictive indication for redo surgery.

The brief clinical management criteria were as follows: patients were managed with a conservative approach, such as, antibiotics, fluid therapy, endoscopic, or radiology intervention and re-laparotomy, according to their condition. If the patient was clinically well and the drain fluid was grossly serous or had a small quantity of bile leakage, active therapeutic intervention was not needed. The abdominal drainage tube was usually removed within 7 days. If the patients were in mild to moderate distress with abdominal pain (and possibly abdominal distension) and the drain fluid had a moderate amount of bile leakage, additional imaging studies (e.g., B-ultrasound or MRCP) should be undertaken. The initial non-operative management of these patients included the administration of intravenous antibiotics, fluid therapy, and/or continued smooth drainage. In some cases, the drainage catheter was removed, even until more than 14 days after surgery. For some of the patients, severe symptoms, such as, abdominal pain and persistent fever (temperature >38.5°C for more than 3 days), developed. Massive amounts of bile in their drains and/or massive ascites under B-ultrasound examination were almost always present. These patients should be subjected to emergency surgical intervention. Otherwise, interventional radiology was first considered in the post-protocol management strategy to control sepsis and treat biliary fistula, allowing inflammation to subside. In a few of the patients, the active emergency surgical intervention was required.

### Data Collection

The patients were assigned to either the pre-protocol group or post-protocol group (before or after March 15, 2013). Preoperative, intraoperative, and postoperative data were collected. Preoperative data such as, demographic characteristics, the preoperative features involved in the disease, etc., were collected. Intraoperative characteristics were collected, including anesthetic, surgical, and transfusion features. The postoperative outcome included surgical and non-surgical outcomes, such as, duration of abdominal drainage tube, postoperative laboratory tests, and/or imaging studies, complication rates, mortality rates, intensive care unit (ICU) admission, redo surgery, and postoperative hospital stay. Follow-up data were recorded by outpatient visits or contact by telephone after discharge. Biliary stricture was determined radiologically with MRI/MRCP.

### Statistical Analysis

The statistical analysis was conducted with SPSS version 22.0 software (SPSS Inc, Chicago, IL). For categorical variables, the data were presented as frequencies with percentages. When a cell of the frequency was <5, Fisher’s exact test was appropriate, otherwise the chi-square test was suitable. Continuous data were described as medians (interquartile ranges) with non-normally distributed data and medians (ranges) with normally distributed data, which were tested with the Mann-Whitney *U*-test and the Wilcoxon rank-sum test or Student’s *t*-test, respectively. In all cases, a *P*-value of < 0.05 was considered statistically significant.

## Results

### Overall Characteristics

During the period prior to the implementation of the strict management protocol (January 1, 2004, to March 15, 2013), there were 1768 patients with a choledochal cyst who underwent surgical management, of these 48 patients suffered from bile leakage (2.7%, 48/1768). Following the implementation of the protocol (March 15, 2013 to March 31, 2020), there were 915 patients who underwent operations and 43 patients who suffered from bile leakage (2.41%, 43/1835). Seven patients were excluded as the medical notes were unobtainable for data extraction. Overall, 84 patients fulfilled the inclusion criteria and were initially included, including 46 patients in the pre-protocol group and 38 patients in the post-protocol group. The baseline characteristics of patients are detailed in Table 1. There were no differences with respect to age, weight, laboratory tests, preoperative ultrasound presentation, etc., As shown in Table 1, patients in the post-protocol group were more likely to have a laparoscopic operation (*P* < 0.001).

**Table 1:** Base characteristics in children with bile leakage following Roux-en-Y hepaticojejunostomy.

**Variable**	**Total population**		
	**Pre protocol (*n* = 46)**	**Post protocol (*n* = 38)**	***p*-Values**
Age (yrs)	2.59 ± 1.94	2.62 ± 1.96	0.50
Female: male	15:31	13:25	0.53
Duration of symptoms before admission, months	2.6 ± 2.3	2.7 ± 2.3	0.29
**Scheduling conditions**			
Emergency (perforated)	19 (41.3)	15 (39.5)	0.52
Elected	27 (58.7)	23 (60.5)	
**Laboratory findings**			
Preoperative anemia (Hb < 12 g/dl), *n* (%)	10 (21.7)	7 (18.4%)	0.46
Preoperative ALB, g/L(normal range: 35–50)	34.9 ± 9.4	35.2 ± 8.7	0.48
Preoperative CRP (mg/L, normal value: 0–8), mean ± SD	11.9 ± 4.7	12.3 ± 4.5	0.39
Preoperative WBC, 109/L, mean ± SD	8.2 ± 5.3	7.9 ± 5.7	0.45
**Ultrasound presentation**			
Mean CBD, cm, mean ± SD	1.5 ± 0.7	1.6 ± 0.7	0.36
**Types of surgery**, ***n*** **(%)**			
Laparoscopic	21 (45.7)	28 (73.7)	
open	25 (54.3)	10 (26.3)	0.008
Operation duration, min	158.6 ± 47.5	169.8 ± 56.4	0.036

### Surgical Interventions

Among the 84 patients with bile leakage, 27 patients underwent redo surgery, including 21 Roux-en-Y hepaticojejunostomy repairs and six drainages of biliary or pancreatic peritonitis. Before 2013, 21(45.7%) patients from the total of 46 pre- protocol patients underwent re-laparotomy in the acute phase according to the corresponding decision of surgeons. The interval time from initial procedure to the redo surgical intervention was quite variable, from 1 to 17 days (median, 3 days). Of the remaining 25 patients (54.3%, 25/46), they experienced spontaneous healing of the leakage, including 13 patients without any interventions and 12 patients with fluid therapy and antibiotics management.

From 2013 onwards, the protocol for surgical intervention was applied more strictly. Of the 38 patients with bile leakage in this period, most of them (84.2%, 32/38) were treated with a conservative approach. Among them, five patients were successfully managed with percutaneous biliary or pancreatic drainage, and seven patients had refractory bile leakage (over 25 days). Only six patients (15.8%, 6/38) were subjected to redo surgery with uncontrollable biliary peritonitis.

### Short and Intermediate Term Outcomes

After implementation of the strict protocol, the surgical interventions (odds ratio [OR] = 4.48 [95% CI, 1.57–12.77]; *p* = 0.003) and ICU admissions (OR = 3.72 [95% CI, 1.11–12.49]; *p* = 0.024) were significantly decreased. Interestingly, the interval from the initial operation to the second operation was longer in the post-protocol group (*p* = 0.048), indicating the longer care window for the redo operation. Furthermore, the overall duration of drainage was not reduced by the implementation of the strict protocol. There was no hospital mortality.

As shown in Table 2, patients within the pre-protocol period were found to have more total complications (10/46, 23.3%) than those with post-protocol implementation (4/38, 9.5%) (*p* = 0.14), including incision dehiscence, surgical site infections, and intraperitoneal abscess, although, there was no significant difference.

**Table 2:** Outcome characteristics in comparison between Pre and Post protocol cohorts.

	**Pre protocol (*n* = 46)**	**Post protocol (*n* = 38)**	***p*-Values**	**Odds ratio (95% CI)**
Surgical interventions, *n* (%)	21 (45.7)	6 (15.8)	0.003	4.48 (1.57–12.77)
Interval from initial operation to redo surgery	3 (1–17)	5 (4-21)	0.048	
No. of patients with complications, *n* (%)	10 (23.3)	4 (9.5)	0.14	
Total number of complications, *n* (%)	12 (57.1)	5 (14.3)	0.12	
ICU admission	14 (32.6)	4 (9.5)	0.024	3.72 (1.11–12.49)
Durations of parenteral nutrition, days	2.7 ± 1.4	3.5 ± 1.6	0.22	
Duration of drainage, days	8.5 ± 3.2	10.1 ± 4.5	0.37	
Postoperative hospital stay, days	8.8 ± 2.6	9.3 ± 2.9	0.35	
Bile duct stricture, *n* (%)	2 (9.5)	1 (0)	0.57	

After 1–3 months, 20 patients were lost to follow-up. The mean follow-up was 1.2 years (1.5–138 months). Bile duct strictures were diagnosed in two patients (2.4%, 2/84) during the long-term follow-up, presenting with jaundice (*n* = 2) and abdominal pain (*n* = 2). Among them, one had a mild stricture and one had severe anastomosis obstruction. One patient was adequately treated by surgical reconstruction. Of the patients with bile duct strictures, there was no difference between them.

## Discussion

The present study revealed that, among patients with bile leakage, a strict surgical intervention strategy exhibited a promising outcome in terms of recovery measures and reductions in redo surgery, as compared with those in the early active surgical intervention. Therefore, it highlights the fact that the strictly surgical intervention may ensure improved patient postoperative recovery.

The experience of the surgical team is one of the most important factors in the management of patients with bile leakage (13, 14). In our institute, the management of bile leakage has evolved after the introduction of the ISGLS definition, preferring a wait-and-see approach (15, 16). We demonstrated that most cases of minor leakage can be treated effectively by conservative management and recovery without complications. Different bile leaks could be managed by different approaches, including endoscopic intervention, biliary drainage, or combinations of these methods (17, 18). Recently, endoscopic treatment, such as, endoscopic sphincterotomy, or nasobiliary drainage, has been in successful in patients with peripheral postoperative bile leakage (19). Pediatric patients with intrahepatic bile duct stones were also successfully managed through the limb of the Roux-en-Y reconstruction with double-balloon enteroscopy (20). The interventional radiology and endoscopic interventions were proven valuable in the present research. On the other hand, the detrimental effect of therapeutic re-laparotomy interventions is presented in the present series, as surgical interventions for bile leakage results in more ICU admissions and surgical complications. Several studies suggested that patients who had undergone operation in the acute phase had more postoperative complications than those in patients operated on in a delayed phase. According to these result, we preferred a radiologic interventional approach to manage the bile leakage.

We also think that, for patients with bile leak, a longer waiting interval is reasonable to allow the abdominal inflammation to subside. The re-laparotomy is the most invasive intervention and technically difficult and often hazardous, with the patient being exposed to a second anesthetic and operative intervention. In the present post-protocol criteria, redo surgery was only considered for patients with sepsis and severe progressive cholangitis, since the principle of surgical intervention was applied more strictly and no extra surgical complications occurred in these patients.

Of course, the patients should be closely monitored for evidence of deterioration after initial management. Severe episodes of cholangitis, jaundice, and intraabdominal sepsis were the indications for immediate intervention, and there should be no delay in endoscopic or surgical intervention once these symptoms develop postoperatively (21, 22). Further, studies should be performed to define the optimal indication for surgical intervention for hepaticojejunostomy. Fortunately, in the current cohort, almost all the patients with cholangitis responded to management with antibiotics. It should be noted that there was no evidence for the use of chronic antibiotic management in patients with bile leakage.

The principle morbidity of concern for bile leakage is biliary anastomotic stricture (23, 24). In early redo surgery, the associated inflammation may lead to biliary stricture because of bile duct ischemia. Because it is the inflammation phase and fibrosis begins, previous reports have suggested that, after the initial 48–72 h, surgical repair of bile duct injury has a high stricture rate. Delayed repair allowed surgeons to wait for a reasonable period of time to demarcate the ischemic damage (25, 26). Fortunately, in our cohort, only a few patients developed postoperative stricture in the post-protocol group, which is less than that reported by others. In our pre-protocol cohort, most of the redo surgeries were performed within the initial 48–72 h. A high postoperative stricture incidence occurred, which was comparable to that of the post-protocol group. Due to the low incidence of this complication, statistical comparison was difficult. Another factor that should be taken into account was the relative short follow-up time period for the post-protocol group, which might somewhat account for the low incidence.

This study has several limitations. First, the retrospective nature of the study may limit the value of our results. Given its low incidence and complex conditions, the nature of bile leakage does not lend itself to a randomized controlled trial. In this retrospective setting, we investigated multiple short- and long-term outcomes, with heterogeneity explored by subgroup in a strict redo surgery protocol. Second, decision-making regarding the surgical intervention was based on each surgeon’s judgment. There should be confounding for the preference of therapy or surgeon’s personal choice. We could not fully adjust for confounding factors, including cholecystectomy features, diagnosis timing, patients’ conditions, and so on. In addition, an optimal information size was not reached regarding several outcomes, owing in part to the rarity of bile leakage, which may result in serious imprecision. Our study should not be interpreted as an arbitrary conclusion that any repair for bile leakage should be delayed. However, to our knowledge, this is the first and largest study contributing to this controversial topic. Further, multicenter collaborative studies are needed to accumulate more robust evidence and to ascertain the optimal timing of intervention under different clinical conditions.

## Conclusion

In summary, the current study provides further evidence that surgical interventions in the acute phase should be strictly performed and a multidisciplinary approach appears to be beneficial in decreasing postoperative complications. As a result, biliary stricture should be considered to assure optimal short- and long-term outcomes following bile leakage.

## Data Availability Statement

The raw data supporting the conclusions of this article will be made available by the authors, without undue reservation.

## Ethics Statement

The studies involving human participants were reviewed and approved by ethics committee of the Children’s Hospital of Chongqing Medical University. Written informed consent to participate in this study was provided by the participants’ legal guardian/next of kin. Written informed consent was obtained from the individual(s), and minor(s)’ legal guardian/next of kin, for the publication of any potentially identifiable images or data included in this article.

## Author Contributions

KZ, LW, CZ, KG, and CG designed the study and analyzed the data. CY, LW, and CZ evaluated the manuscript. CG and KG performed the statistical measurements and analyzed the data. CG analyzed the data and wrote the paper. All authors have read and approved the final manuscript as submitted and agree to be accountable for all aspects of the work.

## Conflict of Interest

The authors declare that the research was conducted in the absence of any commercial or financial relationships that could be construed as a potential conflict of interest.
